# Proliferation of SARS-CoV-2 B.1.1.7 Variant in Pakistan-A Short Surveillance Account

**DOI:** 10.3389/fpubh.2021.683378

**Published:** 2021-05-31

**Authors:** Massab Umair, Muhammad Salman, Zaira Rehman, Nazish Badar, Qasim Ali, Abdul Ahad, Aamer Ikram

**Affiliations:** Department of Virology, National Institute of Health, Islamabad, Pakistan

**Keywords:** upsurge, SARS-CoV-2, B1.1.7, Pakistan, mutation

## Abstract

The emergence of a more transmissible variant of SARS-CoV-2 (B1. 1.7) in the United Kingdom (UK) during late 2020 has raised major public health concerns. Several mutations have been reported in the genome of the B.1.1.7 variant including the N501Y and 69-70deletion in the Spike region that has implications on virus transmissibility and diagnostics. Although the B.1.1.7 variant has been reported by several countries, only three cases have been reported in Pakistan through whole-genome sequencing. Therefore, the objective of the study was to investigate the circulation of B.1.1.7 variant of concern (VOC) in Pakistani population. We used a two-step strategy for the detection of B.1.1.7 with initial screening through TaqPath^TM^ COVID-19 CE-IVD RT-PCR kit (ThermoFisher Scientific, Waltham, US) followed by partial spike (S) gene sequencing of a subset of samples having the spike gene target failure (SGTF). From January 01, 2021, to February 21, 2021, a total of 2,650 samples were tested for SARS-CoV-2 and 70.4% (*n* = 1,867) showed amplification of all the 3 genes (ORF, N, and S). Notably, 29.6% (n=783) samples have been SGTF that represented numbers from all the four provinces and suggest a rather low frequency during the first 3 weeks of January (*n* = 10, *n* = 13, and *n* = 1, respectively). However, the numbers have started to increase in the last week of January, 2021. During February, 726 (93%) cases of SGTF were reported with a peak (*n* = 345) found during the 3rd week. Based on the partial sequencing of SGTF samples 93.5% (*n* = 29/31) showed the characteristic N501Y, A570D, P681H, and T716I mutations found in the B.1.1.7 variant. In conclusion, our findings showed an upsurge of B.1.1.7 cases in Pakistan during February, 2021 affecting 15 districts and warranting large scale genomic surveillance, strengthening of laboratory network and implementation of appropriate control measures in the country.

## Introduction

The last few months of 2020 have witnessed the emergence of different variants of severe acute respiratory syndrome coronavirus 2 (SARS-CoV-2) having characteristic mutations in their genome (1). One such variant is the B.1.1.7 (VOC 202012/01) which was first detected in the United Kingdom (UK) during November 2020, rapidly becoming one of the most prevalent forms of SARS-CoV-2 in the country. Due to its high transmissibility rate (about 50–70%) the variant has now spread to 93 countries as of February 21, 2021 (2, 3).

The B.1.1.7 harbors 17 amino acid changes mostly in the Spike (S) protein (69–70del, 144del, N501Y, A570D, P681H, T716I, S982A, and D1118H) (4). Mutations in the S protein of SARS-CoV-2, which is involved in viral entry into host cells can potentially impact virus transmissibility, diagnostic capability, and pathogenesis. Of particular note is the N501Y change in the receptor binding domain (RBD) that is directly involved in interactions with the human angiotensin-converting enzyme 2 (hACE2) receptor (5). The N501Y mutation can lead to enhanced viral binding efficiency with hACE2 thus rendering the virus more transmissible (6, 7). Similarly, the 69–70 deletion (69–70del) in the S gene may increase cell infectivity and lead to diagnostic failure in real-time PCR amplification kits targeting this region (8). One such assay is the TaqPath^TM^ COVID-19 (ThermoFisher Scientific, Waltham, US) kit that targets three genes of the virus including the S gene. With this method, the 69–70del in the S gene of SARS-CoV-2 results in the spike gene target failure (SGTF) on real-time PCR that has been used as a proxy for the detection of B.1.1.7 variant (9, 10). A recent analysis of the US Food and Drug Administration (FDA) also showed that SGFT on TaqPath^TM^ COVID-19 (ThermoFisher Scientific, Waltham, US) kit can be used as a signal for early identification of 69–70del that can be further investigated to track variant strains (11).

Currently, the genomic surveillance of SARS-CoV-2 in Pakistan is limited to few laboratories that have specialized facilities and expertise, resulting in limited assessment of the introduction, geographic spread and community transmission of SARS-CoV-2 variants such as the B.1.1.7. As of February 26, 2021, only three cases of B.1.1.7 have been reported from Pakistan through whole-genome sequencing by the National Institute of Health (12). We aimed to investigate the detection of B.1.1.7 in Pakistan using the SGTF method followed by confirmation of a subset of samples through partial sequencing of S gene, encompassing the receptor binding domain (RBD).

## Methods

### Sampling

Oropharyngeal samples (*n* = 2,650) collected from patients suspected of COVID-19 and received at the Department of Virology, National Institute of Health, Islamabad was included in the study. The study design was approved by the Internal Review Board of National Institute of Health and the databases were anonymized and free of personally identifiable information.

### RNA Extraction and RT-qPCR

RNA was extracted from the samples using MagMAX™ Viral/Pathogen Nucleic Acid Isolation Kit and KingFisher ^TM^ Flex Purification System (ThermoFisher Scientific, US). For the detection of SARS-CoV-2, TaqPath^TM^ COVID-19 CE-IVD RT-PCR kit (ThermoFisher Scientific, Waltham, US) that targets three genes (ORF1ab, N, and S) was used.

### Sequencing

A subset of samples (*n* = 31/783) with Spike Gene Target Failure (SGTF) were selected for the partial sequencing of the spike gene (S). The majority of the samples sequenced (*n* = 18/31; 58%) were selected from Islamabad due to the highest number of SGTF cases (*n* = 679/783) detected from the city. The remaining samples were sequenced based on their geographic location i.e., representing the three provinces along with the AJK region of Pakistan. The RT-PCR was carried out using the Qiagen^TM^ OneStep RT-PCR (Qiagen, Germany) kit according to the manufacturer's instructions. The primers used for the partial sequencing of the S gene had been reported previously (12). The following reaction conditions were used for the amplification of spike gene fragment: 50°C for 30 min, 95°C for 15 min followed by 40 cycles of 94°C for 1 min, 55°C for 30 s and 72°C for 1 min. PCR was completed with a final extension at 72°C for 10 min. The amplified product was sequenced using the BigDye^TM^ Terminator v3.1 Cycle Sequencing kit on ABI3500xL genetic analyzer (ThermoFisher Scientific, US).

### Phylogenetic Analysis

For phylogenetic analysis, closely related sequences to the study strains were downloaded from GISAID using BLAST (13). Additionally, representative B.1.1.7 sequences from neighboring countries were also included in the analysis followed by multiple sequence alignment using MAFTT. The phylogenetic tree was constructed using BEAST to estimate divergence times. The GTR substitution model (14) model was identified using jModelTest (15) as the best-fit model for phylogenetic analyses with this dataset. For this dataset, BEAST analysis was run for 5 million generations to achieve convergence of parameters by calculating the effective sample size (ESS > 200) using TRACER version 1.6 and tree was visualized *via* Figtree v1.4.2. All the sequences generated in the current study are submitted to the GISAID under the accession numbers: (EPI_ISL_1072998, EPI_ISL_1073001, EPI_ISL_1073004, EPI_ISL_1073012, EPI_ISL_1073015, EPI_ISL_1073021, EPI_ISL_1073023 - EPI_ISL_1073028, EPI_ISL_1073030 - EPI_ISL_1073033, EPI_ISL_1173510, EPI_ISL_1173257, EPI_ISL_1173513, EPI_ISL_1173514, EPI_ISL_1173505, EPI_ISL_1173507, EPI_ISL_1250319 and EPI_ISL_1250574).

## Results

**Figure 1:**
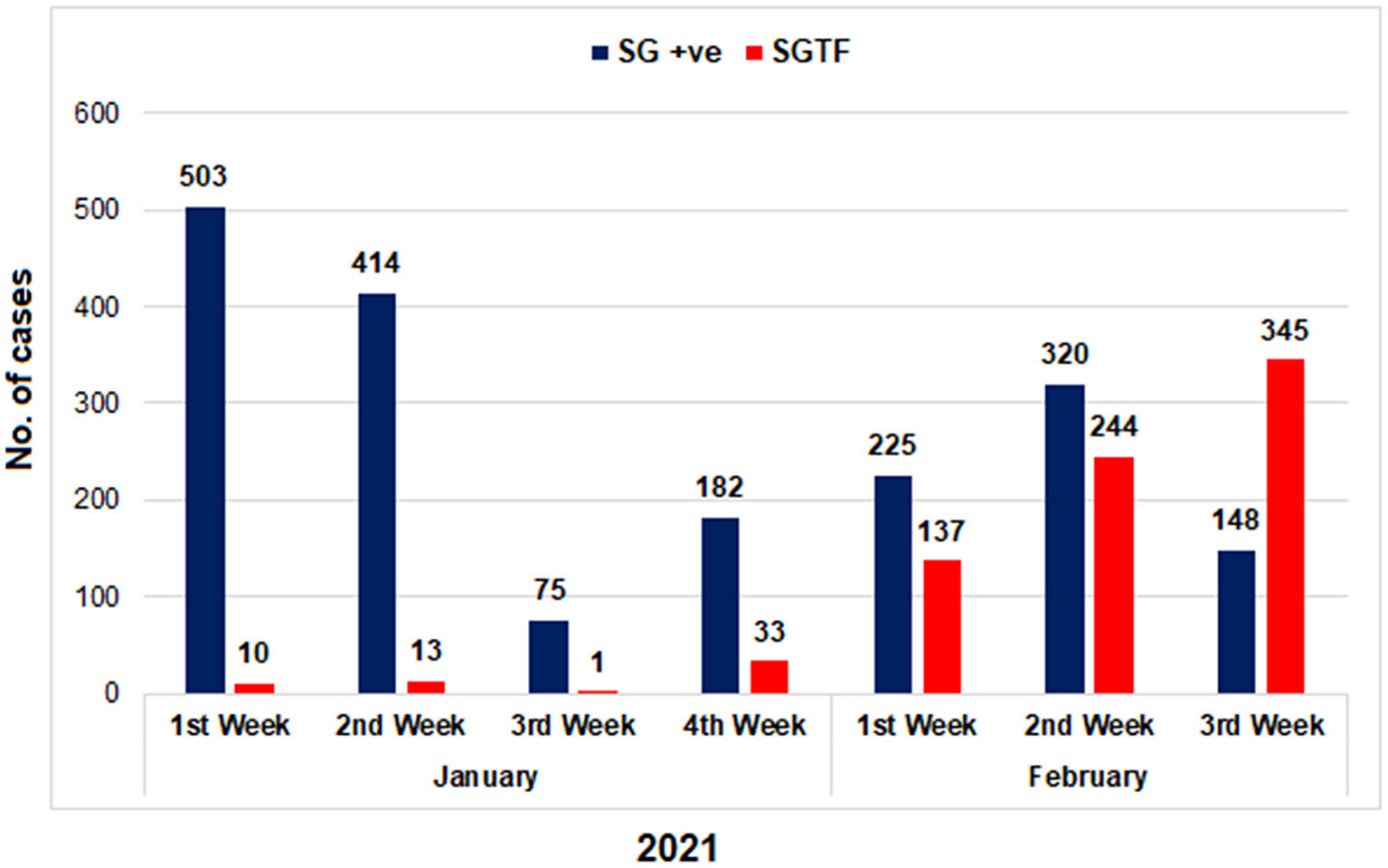
The distribution of COVID-19 patients according to the Δ69–70 deletion during January and first 3 weeks of February, 2021. TaqPath RT-PCR kit was used for detection of SGTF in SARS-CoV-2 patients. X-axis representing the months while Y-axis representing the number of cases. SG +ve: spike gene detection, SGTF: spike not detected.

**Figure 2:**
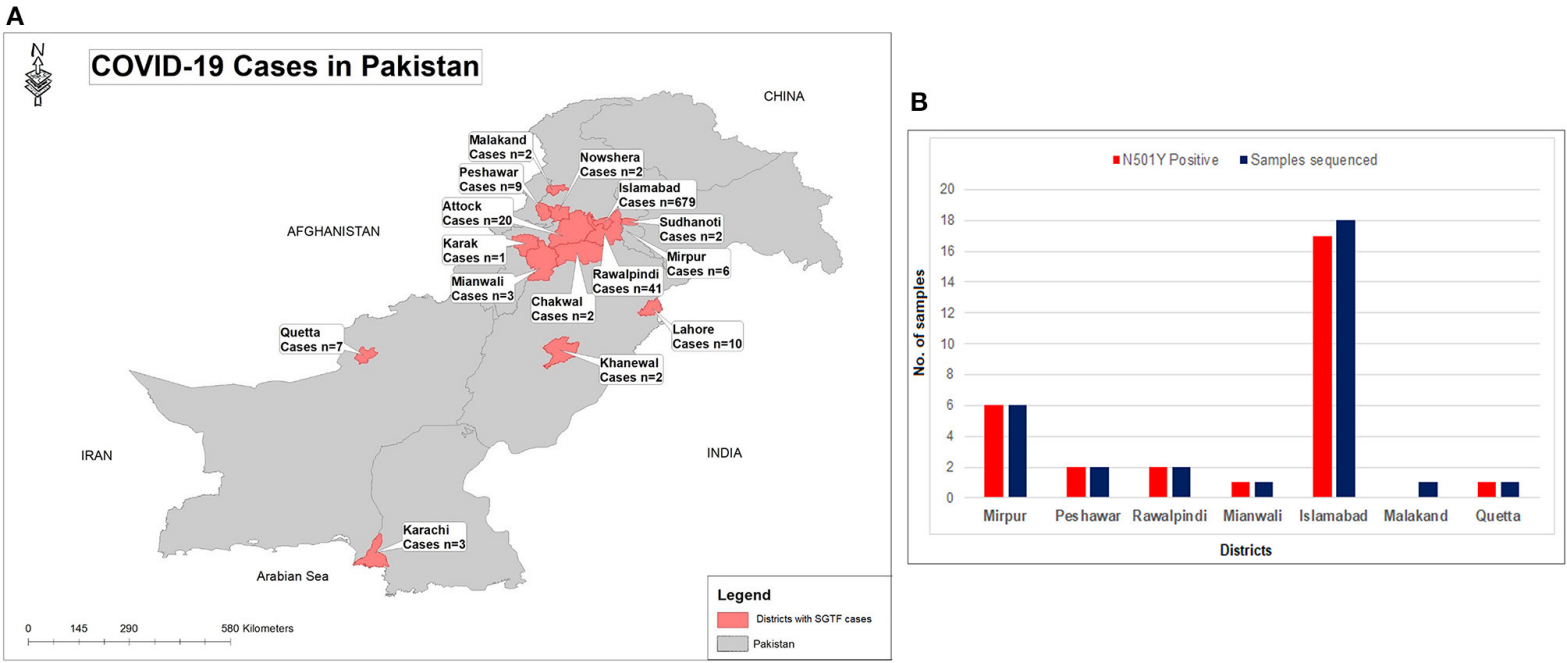
**(A)** Geographical distribution of SGTF cases of SARS-CoV-2 in Pakistan from January- February 21, 2021. **(B)** District wise distribution of SGTF cases which are sequenced and having N501Y mutation. X-axis representing the districts while Y-axis representing the number of cases.

From January 01, 2021, to February 21, 2021, a total of 2,650 samples were tested on real-time PCR for the presence of SARS-CoV-2 using the TaqPath^TM^ COVID-19 CE-IVD RT-PCR kit (ThermoFisher Scientific, Waltham, US). Among these, 70.4% (*n* = 1,867) showed amplification of all the 3 genes (ORF, N, and S), whereas 29.6% (*n* = 783) samples had the spike gene target failure (SGTF). The SGTF cases were detected at a low frequency during the first 3 weeks of January (*n* = 10, *n* = 13, and *n* = 1, respectively), and increased after that. In February, 726 (93%) SGTF cases were reported, with a peak (*n* = 345) during the 3rd week (Figure 1). The majority of the SGTF cases were reported from Islamabad (*n* = 679; 87%) followed by Rawalpindi (*n* = 35; 4.4%) and Attock (*n* = 20; 2.5%). Overall, the variant was detected from 15 districts representing all the four provinces of Pakistan. The distribution of cases among the different districts of Pakistan is shown in Figure 2A.

**Figure 3:**
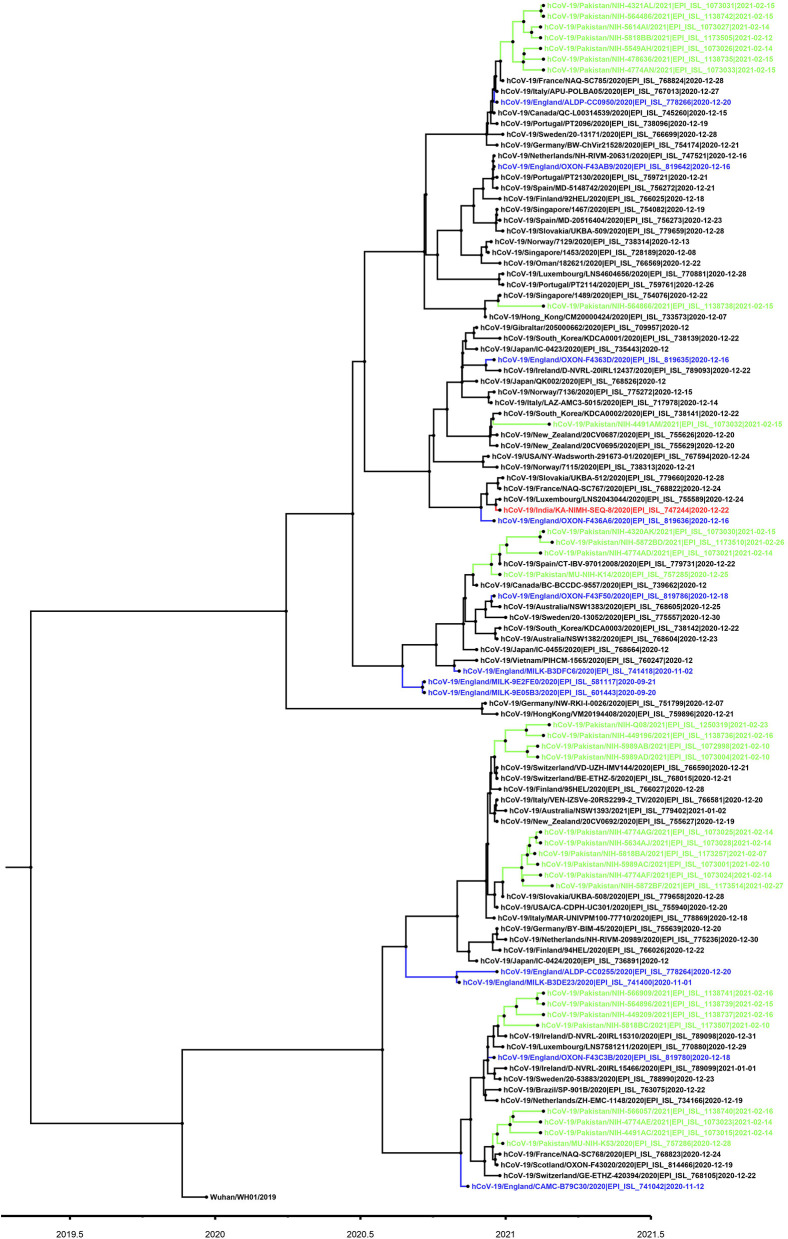
Phylogenetic tree of B.1.1.7 variant viruses (partial S-gene). For phylogenetic analysis, Wuhan reference SARS-CoV-2 (EPI_ISL_529213) sequence was used. The Pakistani sequences are highlighted in green color and blue color shows sequences reported from England. A timescale for evolution of strain is shown at the bottom of tree.

Based on the partial sequencing of spike gene of SGTF samples, 93.5% (*n* = 29/31) showed the characteristic N501Y, A570D, P681H, and T716I mutations found in the B.1.1.7 variant. Sample NIH-4474AE had unique mutations (K444R and V445L) and NIH-5818BB showed a novel mutation H655P in the S protein (Table 1). Two samples (NIH-4490AB and NIH-5872BE) did not show characteristic amino acid changes of B.1.1.7. The age of patients infected with the N501Y variant ranged from 12 to 90 years with a median age of 32.5 years. The female to male ratio is 3:2 where 60% of females and 40% of males were infected. The majority of patients from the study sample pool have reported the common COVID-19 symptoms of fever, headache, bodyache, cough and fatigue whereas only 3 patients reported being asymptomatic (Table 1). Most of the cases have been reported from Islamabad followed by neighboring areas such as Rawalpindi, Mirpur & Peshawar. The distribution of N501Y positive cases according to a geographic area is shown in Figure 2B. Based on phylogenetic analysis, Pakistani strains showed close sequence similarities with the B.1.1.7 variants reported from England, France, Switzerland, Slovakia, Ireland, Spain, New Zealand, Canada and Singapore (Figure 3).

**Table 1:** Clinico-pathological features of the patients along-with the amino acid mutations found in the spike gene of study enrolled samples.

**Sr. No**.	**Lab ID**	**GISAID Accession ID**	**Date of sample collection**	**Age (years)**	**Gender**	**Sign & symptoms**	**Travel history**	**Outcome**	**Location/District**	**Mutations**
1	NIH-4491AC	EPI_ISL_1073015	14-Feb	35	M	Fever & cough	None	Recovered	Islamabad	N501Y, A570D, D614G, P681H, I716T
2	NIH-4774AD	EPI_ISL_1073021	14-Feb	90	F	Fever & cough	None	Expired	Islamabad	N501Y, A570D, D614G, P681H, I716T
3	NIH-4774AE	EPI_ISL_1073023	14-Feb	40	F	Fever, cough, fatigue, headache, loss of taste and smell	None	Recovered	Islamabad	K444R, V445L, N501Y, A570D, D614G, P681H, I716T
4	NIH-4774AF	EPI_ISL_1073024	14-Feb	15	F	Asymptomatic	None	Recovered	Islamabad	N501Y, A570D, D614G, P681H, I716T
5	NIH-4774AG	EPI_ISL_1073025	14-Feb	34	M	Loss of taste and smell	None	Recovered	Rawalpindi	N501Y, A570D, D614G, P681H, I716T
6	NIH-5549AH	EPI_ISL_1073026	14-Feb	27	F	Asymptomatic	Afghanistan	Recovered	Islamabad	N501Y, A570D, D614G, P681H, I716T
7	NIH-5614AI	EPI_ISL_1073027	14-Feb	45	F	Fever, cough, body aches	None	Recovered	Mianwali	N501Y, A570D, D614G, P681H, I716T
8	NIH-5634AJ	EPI_ISL_1073028	14-Feb	44	F	Fever, cough, sore throat, fatigue, loss of taste and smell, body aches	None	Recovered	Islamabad	N501Y, A570D, D614G, P681H, I716T
9	NIH-4320AK	EPI_ISL_1073030	15-Feb	29	F	Fever & body aches	None	Recovered	Islamabad	N501Y, A570D, D614G, P681H, I716T
10	NIH-4321AL	EPI_ISL_1073031	15-Feb	28	M	Fever & body aches	None	Recovered	Islamabad	N501Y, A570D, D614G, P681H, I716T
11	NIH-4491AM	EPI_ISL_1073032	15-Feb	26	F	Fever, cough, sore throat, fatigue, body aches	None	Recovered	Rawalpindi	N501Y, A570D, D614G, P681H, I716T
12	NIH-4774AN	EPI_ISL_1073033	15-Feb	30	F	Fever, cough, fatigue	None	Recovered	Islamabad	N501Y, A570D, D614G, P681H, I716T
13	NIH-5989AB	EPI_ISL_1072998	10-Feb	38	M	Fever & cough	England	Recovered	Mirpur	N501Y, A570D, D614G, P681H, I716T
14	NIH-5989AC	EPI_ISL_1073001	10-Feb	43	M	Fever, cough & headache	England	Recovered	Mirpur	N501Y, A570D, D614G, P681H, I716T
15	NIH-5989AD	EPI_ISL_1073004	10-Feb	43	M	Fever & cough	None	Recovered	Mirpur	N501Y, A570D, D614G, P681H, I716T
16	NIH-478636	EPI_ISL_1138735	15-Feb	31	F	Fever	None	Recovered	Islamabad	N501Y, A570D, D614G, P681H, I716T
17	NIH-449196	EPI_ISL_1138736	16-Feb	28	M	Fever, cough, sore throat, fatigue, headache, loss of taste and smell	None	Recovered	Islamabad	N501Y, A570D, D614G, P681H, I716T
18	NIH-449209	EPI_ISL_1138737	16-Feb	29	F	Fever, cough, sore throat, fatigue, headache, loss of taste and smell, bodyaches	None	Recovered	Islamabad	N501Y, A570D, D614G, P681H, I716T
19	NIH-564866	EPI_ISL_1138738	15-Feb	33	F	Fever and cough	None	Recovered	Islamabad	N501Y, A570D, D614G, P681H, I716T
20	NIH-564896	EPI_ISL_1138739	15-Feb	23	F	Fever, cough, sore throat, fatigue, headache, loss of taste and smell, body aches	None	Recovered	Islamabad	N501Y, A570D, D614G, P681H, I716T
21	NIH-566057	EPI_ISL_1138740	16-Feb	26	M	Fever, cough, fatigue, body aches	None	Recovered	Islamabad	N501Y, A570D, D614G, P681H, I716T
22	NIH-566909	EPI_ISL_1138741	16-Feb	12	F	Fever& bodyaches	None	Recovered	Islamabad	N501Y, A570D, D614G, P681H, I716T
23	NIH-564486	EPI_ISL_1138742	15-Feb	65	M	Fever, cough, sore throat, headache	None	Recovered	Islamabad	N501Y, A570D, D614G, P681H, I716T
24	NIH-5818BA	EPI_ISL_1173257	7-Feb	34	F	Fever & headache	None	Recovered	Mirpur	N501Y, A570D, D614G, P681H, I716T
25	NIH-5818BB	EPI_ISL_1173505	12-Feb	31	F	Asymptomatic	England	Recovered	Mirpur	N501Y, A570D, D614G, H655P, P681H, I716T
26	NIH-5818BC	EPI_ISL_1173507	10-Feb	32	M	Fever & cough	America	Recovered	Mirpur	N501Y, A570D, D614G, P681H, I716T
27	NIH-5872BD	EPI_ISL_1173510	26-Feb	50	F	Fever, cough, headache & body aches	None	Recovered	Peshawar	N501Y, A570D, D614G, P681H, I716T
28	NIH-5872BF	EPI_ISL_1173514	27-Feb	60	F	Fever & cough	None	Recovered	Peshawar	N501Y, A570D, D614G, P681H, I716T
29	NIH-Q08	EPI_ISL_1250319	23-Feb	21	M	Fever & cough	None	Recovered	Quetta	N501Y, A570D, D614G, P681H, I716T

## Discussion

As of March 10, 2021, 144,988 (30% prevalence) sequences of the B.1.1.7 lineage have been detected worldwide with the majority of cases reported in the UK (*n* = 113,593; 53% prevalence). High number of B.1.1.7 infections has been reported from other countries such as Denmark (*n* = 4,889), Germany (*n* = 4,475), Italy (*n* = 2,371), Ireland (*n* = 2,262), Switzerland (*n* = 2,165), Belgium (*n* = 2,118), France (*n* = 1,946), Netherlands (*n* = 1,645) and Spain (*n* = 1,444) (16). Pakistan is the fifth most populous country in the world with 595,239 cases and 13,324 deaths reported due to COVID-19 until March 10, 2021. The most affected province is Sindh (*n* = 259,956), followed by Punjab (*n* = 178,648) and KP (*n* = 74,167); notably, the health system of the capital city Islamabad has been considerably challenged with a high number of cases (*n* = 45,976) (17). Although the COVID-19 diagnostic facilities have been extended to 165 laboratories with an average testing capacity of 61,000 and daily testing around 35,000, limited data is available for the genomic diversity of SARS-CoV-2 from the country, with only 58 whole-genome sequences deposited on GISAID as of March 10, 2021. Among the VOCs only 3 cases of B.1.1.7 variant have been reported from Pakistan (12) mainly due to the lack of a genomic surveillance network that can detect these variants in real-time. Moreover, since January 05, 2021, no whole-genome sequence data of SARS-CoV-2 strains from the country have been deposited on GISAID or NCBI. Therefore, molecular assays that can detect the B.1.1.7 and other VOCs in addition to the whole-genome sequencing becomes invaluable to countries like Pakistan during the pandemic.

In the current study, we found a sharp rise in SGTF samples using TaqPath^TM^ COVID-19 CE-IVD RT-PCR kit (ThermoFisher Scientific, Waltham, US) during February 2021, an indicator of the rapid spread of B.1.1.7 variant in the indigenous population. The use of SGTF as an early indication for the detection of B.1.1.7 has been successfully used in the UK where more than 97% of cases were confirmed to be the B.1.1.7 variant (7). Although the SGTF provides a practical option for the screening of the B.1.1.7 variant particularly in the resource-poor settings where genomic surveillance is not possible, it is not definitive for B.1.1.7 (18). Therefore, we used partial sequencing of the S gene on a subset of SGTF samples as a confirmatory method for B.1.1.7 variant detection. The sequencing results confirmed 93% (*n* = 29/31) cases as B.1.1.7, having the marker S protein mutations (N501Y, A570D, P681H, and T716I). Notably, two SGTF samples (NIH-4490AB and NIH-5872BE) showed no such mutations whereas sample NIH-4774AE had two rare mutations K444R and V445L. According to GISAID data (March 10, 2021), the amino acid change K444R has been detected in 36 sequences from 5 countries and the last strain with such a mutation was reported from England (EPI_ISL_777390) in December 2020. Similarly, the V445L change has only been reported once in a strain collected on December 28, 2020 from Denmark (hCoV-19/Denmark/DCGC-24535/2020; EPI_ISL_795176). We also found a novel mutation H655P (655Histidine > Proline) in one of our study sample NIH-5818BB (EPI_ISL_1173505). The amino acid residue 655 is located near the cleavage site, existing between the receptor binding domain (RBD) and the fusion peptide, and has been hypothesized to play a role in regulating spike glycoprotein fusion efficiency (19, 20). Our study did not evaluate the functional impact of H655P mutation on the fusion efficiency of spike protein, however, highlights the importance of continuous monitoring of amino acid changes in the spike region of circulating strains. Previously, H655Y (655Histidine > Tyrosine) amino acid change has been shown to confer escape from monoclonal antibodies in cell culture systems (19).

Our findings of the detection and increased prevalence of highly transmissible B.1.1.7 variant cases gives a plausible reason behind the recent upsurge of COVID-19 cases observed at the national level. In Islamabad from where most of the SGTF cases were reported during the current study, the average cases per day (*n* = 235) have more than doubled in March (until 11th) as compared to January and February 2021 (*n* = 111 and *n* = 106, respectively). A similar trend has been reported nationwide where on March 10, 2021, more than 2,000 infections (positivity = 5.3%) were reported since January 29, 2021, a sign of a possible start of the third wave in the country (21). Although the increased transmissibility of B.1.1.7 VOC has been reported (6, 7) there has been much concern about the effect of this variant on disease severity. Preliminary assessment of disease severity by Public Health England (PHE) showed an insignificant difference in the risk of hospitalization or death in people infected with B.1.1.7 vs. infection with other variants (22). More recently, the New and Emerging Respiratory Virus Threats Advisory Group (NERVTAG) presented evidence to the Strategic Advisory Group of Experts on Immunization (SAGE) of increased disease severity in people infected with B.1.1.7 compared to those infected with non-VOC virus variants (unpublished data). In the current study, one of the patients infected with the B.1.1.7 variant died (*n* = 1/28; 3.5%) whereas 5 (17.8%) were hospitalized. Although our study did not focus on the effect of B.1.1.7 on disease severity, the spread of this variant to different geographic locations of the country with an increasing prevalence has the potential to cause additional mortality in Pakistan (23).

In an attempt to curb the disease, Pakistan started its vaccination campaign on February 02, 2021, with a two-dose inactivated virus vaccine called the Sinopharm. Among the first batch of 0.5 million doses received, the country's frontline health workers will be vaccinated in the first phase followed elderly population aged ≥ 60 years. In addition, Pakistan has secured 17 million doses of AstraZeneca (viral vector) vaccine for the first half of 2021 through COVAX. Although the Sinopharm vaccine has demonstrated an efficacy of 79% in clinical trial (24), the efficacy against the B.1.1.7 VOC is unknown. On the other side, AstraZeneca has shown similar efficacy (74.6%) against the B.1.1.7 as other SARS-CoV-2 lineages (25). With the emergence and rapid spread of B.1.1.7 variant in Pakistan highlighted from our findings it is critical for the health authorities to make the appropriate decisions in the selection of suitable interventions for the control of the disease. Moreover, it will guide health authorities to take appropriate control measures to prevent the further spread of the variant in the country.

The small sample size and representation of a limited geographic area is a major limitation of our study. However, our findings provide an early indication of the spread of the B.1.1.7 variant in indigenous population and warrants large-scale monitoring across the country. Moreover, it serves as a baseline for future studies focusing on exploring the molecular epidemiology/genomic diversity of B.1.1.7 VOC in Pakistan. The emergence of B.1.1.7 lineage with S:E484K cases in the UK followed by its detection in other countries highlights the need for continuous surveillance in Pakistan.

In conclusion our study findings provide a preliminary evidence of the spread of the B.1.1.7 variant in indigenous Pakistani population with partial spike (S) gene sequencing of samples having the spike gene target failure (SGTF). The study demonstrated rise of SGTF cases in February containing characteristic B.1.1.7 spike mutations potentially suggesting a high prevalence and therefore, warrants large-scale monitoring across the country. Moreover, it highlights the hotspot areas affected by the B.1.1.7 and guides district health authorities to take appropriate measures including smart lockdowns to curb the disease. The study also demands for more stringent surveillance and identification of other SARS-CoV-2 variants which can emerge indigenously or exported from other regions.

## Data Availability Statement

The raw data supporting the conclusions of this article will be made available by the authors, without undue reservation.

## Ethics Statement

The studies involving human participants were reviewed and approved by Institutional review board, National Institute of Health, Islamabad, Pakistan. The patients/participants provided their written informed consent to participate in this study.

## Author Contributions

MU, MS, and AI contributed to conception and design of the study. NB, QA, and AA involved in sample processing. MU and ZR performed the analysis and wrote the manuscript. All authors contributed to the article and approved the submitted version.

## Conflict of Interest

The authors declare that the research was conducted in the absence of any commercial or financial relationships that could be construed as a potential conflict of interest.
